# Mayer-Rokitansky-Küster-Hauser syndrome with 22q11.21 microduplication: a case report

**DOI:** 10.1186/s13256-021-02716-6

**Published:** 2021-04-21

**Authors:** Domenico Dell’Edera, Arianna Allegretti, Mario Ventura, Ludovica Mercuri, Angela Mitidieri, Giacinto Cuscianna, Annunziata Anna Epifania, Elisena Morizio, Melissa Alfonsi, Paolo Guanciali-Franchi

**Affiliations:** 1 Medical Genetics Unit, “Madonna delle Grazie” Hospital, Contrada Cattedra Ambulante, Snc, 75100 Matera, Italy; 2 Department of Biology, University of Bari, Bari, Italy; 3 Unit of Diagnostic Radiology and General Interventional Radiology, “Madonna delle Grazie” Hospital, Matera, Italy; 4 Department of Medical Genetics, ‘G. D’Annunzio’ University, Chieti, Italy; 5 Medical Genetics Unit, SS Annunziata Hospital, Chieti, Italy

**Keywords:** Mayer-Rokitansky-Küster-Hauser syndrome, Müllerian anomalies, Multiple congenital anomalies, 22q11.2 microduplication

## Abstract

**Background:**

Mayer-Rokitansky-Küster-Hauser (MRKH) syndrome (Online Mendelian Inheritance in Man [OMIM] #277000) is a congenital condition characterized by the total or partial agenesis of vagina and uterus. Agenesis can be isolated (MRKH 1) or associated with other renal, vertebral or cardiac defects (MRKH 2).

**Case presentation:**

In this paper, we report a case of a Caucasian patient showing the clinical signs associated with MRKH. Array-based comparative genomic hybridization (a-CGH) analysis revealed a microduplication of approximately 3.01 megabases (Mb) located on the long arm of chromosome 22 (22q11.21). Microduplications affecting the 22q11.21 region have been shown to be associated with MRKH syndrome and Müllerian aplasia. The phenotype of patients with 22q11.2 duplication (OMIM #608363) appears extremely variable, ranging from apparently normal to mild learning difficulties or with multiple defects, sharing features with DiGeorge/velocardiofacial (DGS/VCFS) syndrome.

**Conclusions:**

The altered gene expression together with other genetic, nongenetic, epigenetic or environmental factors can cause the extremely variable phenotype in patients carrying such duplication. Therefore, we can consider MRKH syndrome to be one of the clinical features of DGS/VCFS syndrome.

## Background

Mayer-Rokitansky-Küster-Hauser (MRKH) syndrome (Online Mendelian Inheritance in Man [OMIM] #277000) is a rare congenital condition (1:5000 newborn girls) characterized by total or partial agenesis of the vagina and uterus, in an otherwise phenotypically normal female with a normal 46,XX karyotype [1]. Agenesis can be isolated (MRKH 1) or associated with extra-genital anomalies such as renal, vertebral or cardiac defects (MRKH 2) (OMIM #601076) [2]. Generally, the first clinical manifestation is primary amenorrhea in patients with a diploid 46,XX karyotype, normal sex hormone level, female ovaries and external genitalia, and secondary sexual characteristics.

Even if most cases are sporadic, familial clustering has been reported, suggesting a genetic cause of the disease. It could be possible that in such cases there is an autosomal dominant inheritance with an incomplete penetrance and variable expression [3].

A clear genetic cause has not yet been identified, even if several microdeletions and microduplications (1q21,1; 2q12.1q14.1; 7p14.3; 16p11.2; 17q12; 22q11.21; 22q11.21q11.23) and point mutations in genes located on these loci (RBM8A, PAX8, TBX1, TBX6, LHX, HNF1B, WNT4) have been identified in some patients [3, 4].

MRKH syndrome has been occasionally reported to be associated with Dandy-Walker malformation, *situs inversus*, Meckel-Gruber syndrome, Holt-Oram syndrome, McKusick-Kaufman syndrome and Bardet-Biedl syndrome, suggesting that in some cases, MRKH can be seen as a ciliopathy [2].

Microduplications affecting the 22q11.21 region have been shown to be associated with MRKH syndrome and Müllerian aplasia in some cases [3, 5, 6].

The 22q11.2 duplication (OMIM #608363) may be inherited in an autosomal dominant manner or as a *de novo* condition with incomplete penetrance [7]. Thus, the phenotype of patients appears extremely variable, ranging from apparently normal to mild learning difficulties or with multiple defects, sharing features with DiGeorge/velocardiofacial (DGS/VCFS) syndrome (OMIM #601362), such as heart defects, neurodevelopmental disorders, intellectual disability, growth delay, hypotonia and unspecified urogenital abnormalities [7, 8]. Such duplication can be found in apparently normal parents of a patient, suggesting that many individuals carrying a 22q11.2 duplication do not show any phenotypic sign [7].

We report a *de novo* chromosomal duplication of the 22q11.21 region in a girl affected by MRKH 1 and mild mental retardation.

## Case presentation

**Fig. 1:**
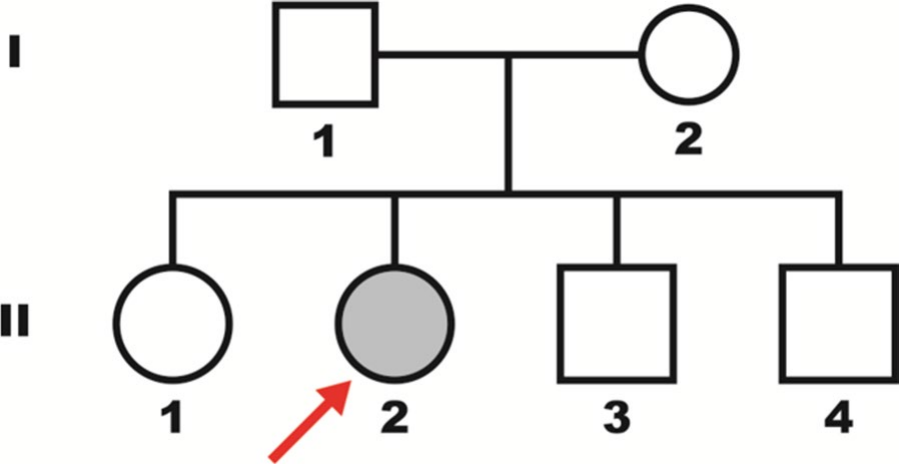
Pedigree of the family. The arrow (red) indicates in the family tree the index case II2 (grey)

**Fig. 2:**
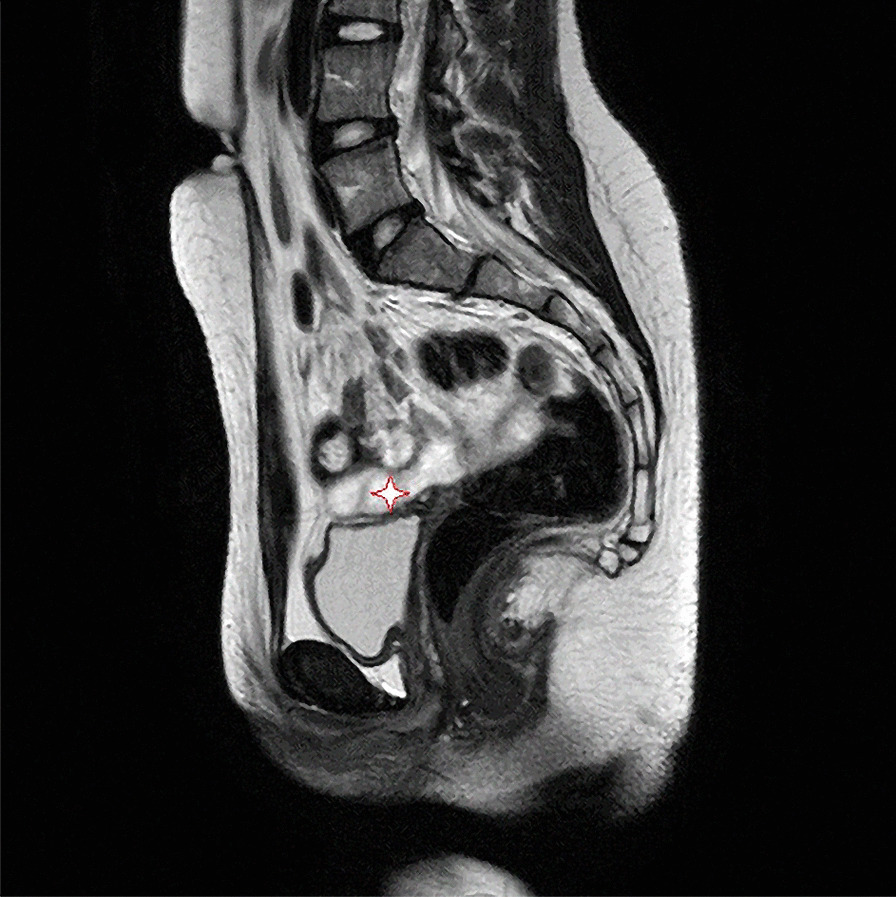
Nuclear magnetic resonance imaging. Medium sagittal image enhanced in T2. The absence of the uterus (star) is confirmed

**Fig. 3:**
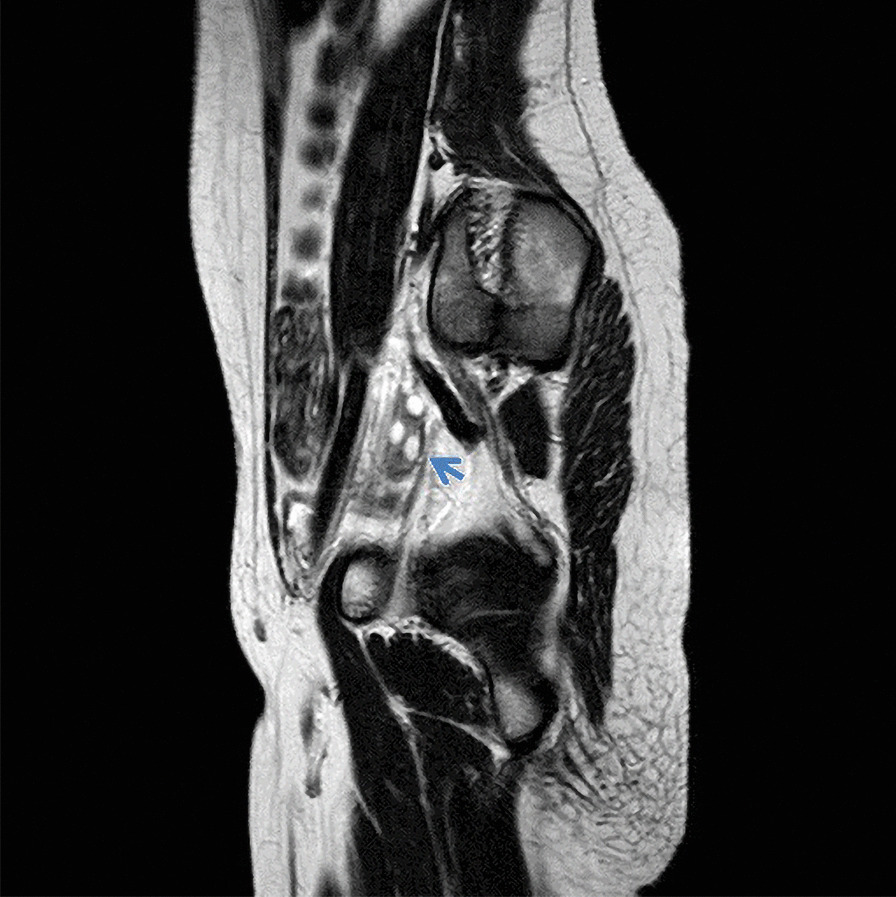
Nuclear magnetic resonance imaging. Paramedian sagittal image enhanced on the left in T2. Left ovary (arrow) is highlighted

The proband was a 15-year-old Caucasian girl, born at 39 weeks of gestation of an uncomplicated pregnancy, as the second child from healthy unrelated parents. She had two unaffected brothers and one sister (Fig1). Parents, brothers and sister showed a normal phenotype. Family history was unremarkable. Between 5 and 7 years of age, she was diagnosed with specific delay in reading skills due to the presence of phonological phonetic disorder. At the age of 15, due to amenorrhea and nonspecific abdominal pain, she underwent standard blood biochemical tests, which showed analyte levels at a normal range. Physical examination showed no particular signs. The girl weighed 42 kg and was 155 cm tall; no heart defects, cleft palate or hearing loss were detected. Ultrasound examination, while showing a normal anatomical structure of the abdominal organs, revealed a hypoplastic uterus (23 mm longitudinal diameter) related to the age of the ovaries. Nuclear magnetic resonance imaging (MRI) confirmed the absence of the uterus and the upper part (2/3) of the vagina, with the presence of thin and nuanced branches of fibrosis (Fig. 2). The rectum and bladder were normally represented. The ovaries were in place, of normal size, with small subcortical follicles (Fig. 3). No signs of brain malformations were detected. Secondary sexual characteristics were normal, as were the external genitalia. Following these clinical features, a diagnosis of MRKH type 1 syndrome was made.

## Methods

Cytogenetic and array-based comparative genomic hybridization (a-CGH) analyses were performed on the peripheral blood of the patient, her parents and her brothers and sister. High-resolution chromosomes were G-bands by Trypsin using Giemsa (GTG) banded using the standard procedure. Genomic DNA was extracted from peripheral blood leukocytes using the QIAGEN QIAamp^®^ DSP DNA Blood Mini Kit (DNA IQ™ system) (Qiagen S.r.l., Milan, Italy), in accordance with the manufacturer’s specifications.

A-CGH analysis was performed using an International Standards for Cytogenomic Arrays (ISCA) Consortium v2 4×180K oligo platform (Oxford Gene Technology), with 25Kb probe spacing (higher resolution in ISCA region). Experiments were conducted according to the manufacturer’s protocol. Commercial reference Deoxyribonucleic acid (DNA) (male and female) provided by Promega G1521 were used for the analysis. The slides were scanned with an InnoScan 710 Microarray Scanner, and captured images were analyzed with CytoSure Interpret software version 4.10. Genomic region analysis was performed according to the human reference sequence hg19GRCh37. The copy number variations (CNVs) found in the proband were compared with genomic variants present on different databases (DECIPHER: https://decipher.sanger.ac.uk; UCSC Genome Browser: https://genome.ucsc.edu; Clinical Genome Resource (Clingen): http://clinicalgenome.org; Troina Database of Human CNVs: http://gvarianti.ho melinux.net/gvariantib37/index.php). *In situ* fluorescence hybridization (FISH) was performed on nuclei and metaphases using a TBX1 probe (Cytocell, ref: LPU 014-S/LPU 014; probe specification: TBX1, 22q11.2, Red N85A3, 22q13.3, Green).

## Results

**Fig. 4:**
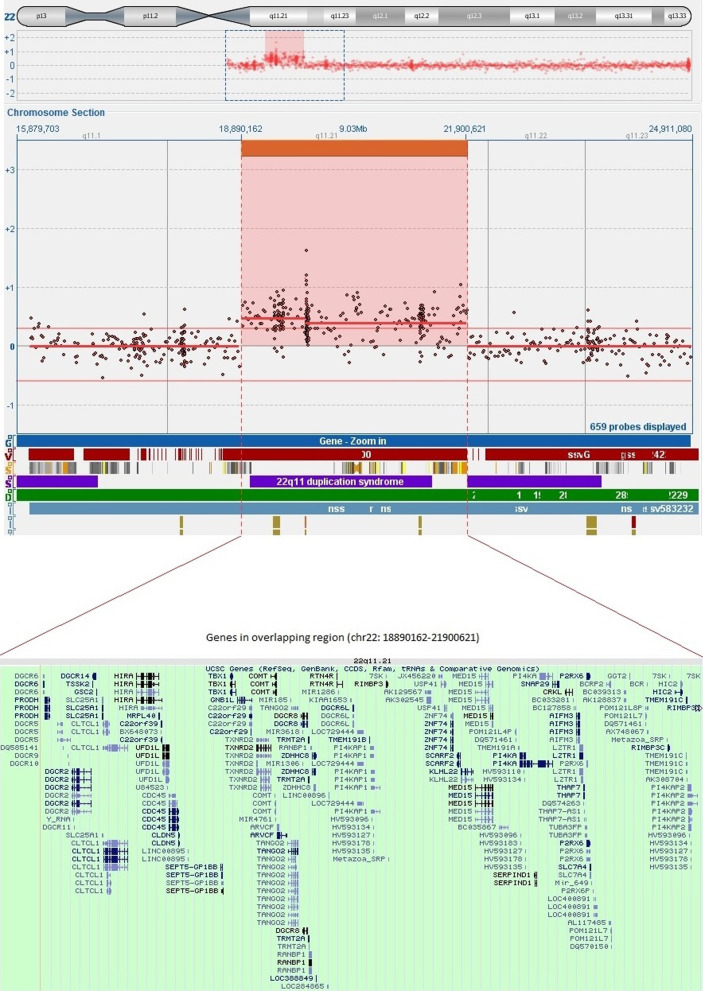
Chromosome 22q11.21 duplication in our patient. The top panel shows the ideogram of chromosome 22 with the 22q11.21 duplicated region marked in a small red box (chr22:18890162-21900621). The scatter plot of the a-CGH data, in the central panel, shows a 3.01 Mb microduplication of the22q11.21 region in our patient. The University of California, Santa Cruz (GRCh37/hg19 assembly) genes in the overlapping region are shown in the bottom panel

Karyotypes of the patient, her parents, and her brothers and sister were normal. The a-CGH analysis performed on the patient detected a *de novo* microduplication of approximately 3.1 megabases (Mb) on chromosome 22q11.21 (chr22:18890162-21900621). Thirteen out of 44 genes located on the duplicated region are OMIM-morbid genes: CDC45, COMT, GP1BB, LZTR1, PIK4A, PRODH, RTN4R, SCARF2, SERPIND1, SLC25A1, SNAP29, TANGO2, TBX1 (Fig. 4). The presence of duplication 22q11.21 was confirmed by FISH analysis. Familial segregation showed that the duplication had arisen *de novo* in the patient.

A 22q11.2 duplication is defined as the presence of a 3Mb (LCR A-D) or 1.5Mb (LCR A-B) proximal tandem duplication. Such duplication can arise *de novo* or may be inherited in an autosomal dominant manner [7].

### Discussion

The phenotype of patients carrying a 22q11.2 duplication is extremely variable, ranging from apparently normal to intellectual/learning disability with or without multiple defects, sharing features with DGS/VCFS such as heart defects, velopharyngeal insufficiency, with or without cleft palate, and unspecified urogenital abnormalities [8]. Ou *et al*. [9] reported three probands with A-D (3 Mb) and two patients with A-B (1.5 Mb) region duplications. In addition, three patients were found to have a copy number gain with a single clone of the a-CGH in the 22q11.2 region. Clinical findings included developmental delay, speech delay, variable dysmorphic features, hypernasal speech, hearing impairment and behavioral abnormalities. In one male patient, initially assigned a female gender at birth, carrying a unique microduplication of about 1 Mb, an undeveloped phallus with a penoscrotal meatus and severe chordee were reported.

Three cases of microduplication on 22q11.22 or on 22q11.21 in association with MRKH syndrome have been reported. The first case is a 3-year-old girl affected by Müllerian aplasia and thrombocytopenia-absent radius (TAR; OMIM #27400) presenting a complete uterus, vagina and ovaries agenesis, with a microduplication of 0.6 Mb, partially overlapping the distal segment of the 22q11.21 microdeletion/microduplication syndrome region. A duplication of approximately 3.5 Mb on the 1q21 region was also found. Such duplication encompasses both the recurrent microdeletion/microduplication (Ensembl Genome Database) and the TAR syndrome susceptibility regions. Both these genomic variants were maternally inherited [1].

The second case was described by Leidig *et al*. [3]. The patient was affected by MRKH 1 with a duplication on the 22q11.21–q11.23 region and a gain of approximately 3.4 Mb. Such duplication partially covers the distal part of the 22q11.21 microdeletion/microduplication region.

The third case is a patient with a partially septate uterus, in which a microduplication of about 2.6 Mb in the 22q11.21 region was reported [6], but no further information was provided on inheritance of such duplication.

In cases of MRKH syndrome, recurrent microdeletions and microduplications (1q21,1; 2q12.1q14.1; 16p11.2; 17p14.3; 17q12; 22q11.21; 22q11.21q11.23) and mutations in genes located on these loci (RBM8A, PAX8, TBX1, TBX6, LHX, HNF1B, WNT4) have been identified [1, 3, 4]. Deletions and duplications of the DGS/VCFS region have also been found in MRKH patients [2]. A 2.6 Mb deletion [1] was reported in a patient showing Müllerian malformation with vaginal agenesis and a rudimentary uterus, mild learning disabilities and mild dysmorphic features. The region containing the TBX1 gene was not deleted. The TBX1 gene is responsible for some of the major clinical features of DGS/VCFS. The authors stated that the presence of the TBX1 gene might be responsible of the mild phenotype showed by the patient [1]. Moreover, Leidig *et al*. [3] reported a small deletion on 22q11.21, not including the TBX1 region, in a patient with MRKH 1 syndrome. Both over- and under-expression of TBX1 can characterize the DGS/VCFS phenotype [10]. New mutations or duplications involving TBX1 have been found in patients without deletion with a DGS/VCFS phenotype [8, 11, 12].

## Conclusion

In conclusion, altered TBX1 expression together with other genetic, nongenetic, epigenetic or environmental factors can cause the extremely variable phenotype in patients carrying a 22q11.2 duplication [8]. Genes other than TBX1 could be involved in the development of uterine malformations described in MRKH syndrome, such as SNAP29 [6], or other genes in the same genetic pathway as TBX1 [13]. Then we can consider MRKH syndrome to be one of the clinical features of DGS/VCFS syndrome.

## Data Availability

Not applicable
